# Clinical estimates of three physiologic capacities explain a majority of unipedal stance time

**DOI:** 10.1007/s40520-025-03164-8

**Published:** 2025-10-07

**Authors:** James K. Richardson, Stephen R. Lord, Kim Delbaere, James A. Ashton-Miller

**Affiliations:** 1 Department of Physical Medicine & Rehabilitation, University of Michigan, 325 E. Eisenhower Pkwy, Suite 200, Ann Arbor, MI 48108 USA; 2 Neuroscience Research Australia, University of New South Wales, Sydney, Australia; 3 Department of Mechanical Engineering, University of Michigan, Ann Arbor, MI USA

**Keywords:** Postural balance, Processing speed, Muscle strength, Proprioception, Clinical medicine

## Abstract

**Background:**

Recent prospective research indicates that unipedal stance time (UST) of < 15 s in middle/older adults increases their risk of repetitive falls within 5 to 10 years.

**Aim:**

To determine the extent that clinical measures of three physiologic capacities, peripheral afferent acuity, processing speed, and proximal frontal plane strength, are responsible for UST.

**Methods:**

UST, distal lower limb clinical vibratory sense, short latency go/no-go accuracy using ReacStick, and lateral plank time, were evaluated in a cohort (*n* = 172, 51% female, age 64.8 +/- 9.6 years) with diabetic neuropathy (*n* = 31), cirrhosis (*n* = 94), and no known neurologic disease (*n* = 47) using age, body mass index (BMI), sex, and medication number as covariates.

**Results:**

Multivariate analyses demonstrated that the three variables separately, and as a composite variable (vibration time + reaction accuracy/2 + lateral plank time), were associated with UST (adjusted R^2^ = 0.66 and 0.65, respectively) for the entire group, and for diabetic neuropathy, cirrhosis, and no known disease groups separately (adjusted R^2^ = 0.59, 0.60, and 0.68, respectively). The composite variable also classified participants into those with UST > and < 15 s (receiver operator characteristics area under the curve (AUC) = 0.92 (95% CI = 0.88, 0.96)).

**Discussion:**

These findings allow clinicians to identify specific physiologic deficits and develop targeted intervention strategies to improve UST.

**Conclusion:**

Clinical estimates of three physiologic capacities predict almost 2/3 of UST variability in middle/older people, rendering age, BMI, sex, and medication number less relevant.

## Introduction

Unipedal stance time (UST) has been recommended as a measure of balance and fall risk in older adults [1]. A recent review suggests that a longer UST identifies those at low risk for falling [2]. Additionally, recent large prospective studies found that people in mid/later life (age 53 years) with a UST of less than 15 s had a three-fold risk of recurrent falls when evaluated 10 to 12 years later as compared to people with UST of 15 s or greater [3]. Additionally, a second large prospective study of people in mid/later-life (61.7 ± 6.8 years) found that participants who could not reliably balance on one foot for ≥ 10 s at baseline were more likely to die over a median follow-up period of seven years [4]. Similarly, UST of less than 15 s was associated with poor locomotor function in mid/later life women (61.8 ± 10.2 years) [5]. Taken together, these studies suggest that diminished UST identified in mid/later life serves as a marker for reduced mobility, recurrent falls and possibly mortality within 5 to 10 years.

However, the physiologic underpinnings of UST are incompletely explored and currently not available to clinicians. In prior work we found that ankle proprioceptive precision and adequate hip strength are required for reliable unipedal stance [6, 7]. Additionally, clinical observation and biomechanical research show that unipedal stance is not static but involves a series of short latency adjustments to maintain a wavering center of mass over the relatively narrow base of support provided by one foot. Analyses have shown that 3 to 5 mediolateral adjustments per second are usual, or one adjustment every 200 to 330 ms, confirming the need for rapid processing speed [8]. Cortical involvement is likely necessary, as declines in unipedal balance performance are evident even in young people under dual task conditions [9]. Accordingly, current understanding of mediators of UST suggests that stable UST requires: (a) Precise lower limb proprioceptive input to rapidly detect subtle shifts of ground reaction forces [10]; (b) The capacity to quickly integrate afferent data and inhibit distractors. and inhibit the usual action of placing the suspended foot on the floor to regain balance [11, 12]; and (c) Intact neuromuscular function in key muscle groups, including ankle invertors/evertors, and most importantly hip abductors [6, 7, 13].

With this background in mind, clinicians need sensitive measures which efficiently detect subtle changes in somatosensory precision, cognitive processing speed, and force generation in key muscle groups in order to determine why, specifically, a person in their mid-late years cannot perform UST for longer than 10–15 s. Measurements must be sufficiently sensitive to allow differentiation between continued optimal function and gradual, often sub-clinical declines. This sensitivity to change contrasts with many clinical assessments which are generally intended to detect disease, rather than subtle departures from previous high-level function. For example, manual muscle testing using the 5-point scale can likely detect reduced strength due to an inflammatory myopathy or femoral neuropathy, but is unlikely to detect the difference between a younger man in his most athletic years and the same man in his middle years who now ascends stairs with less ease. Similarly, most cognitive measures evaluate cognitive impairments related to letters, words, and numbers over intervals of seconds (e.g., Trails A/B) rather than the ability to respond within a 350-millisecond window as is necessary to successfully recover from a trip [14]. We hypothesize that subtle decrements in one or a combination of these three critical physiologic capacities occur in some people in their middle years leading to decreased UST, which in turn serves as a marker for repetitive falls. To explore the concept that UST is predictable by clinically quantifying relevant underlying physiologic capacities we analyzed data which included our best clinical estimates, based on prior research, of somatosensory precision, cognitive processing speed, and frontal plane force generation. The underlying research question is whether, and to what extent, these three clinical measures can predict UST as a continuous variable and, also, as a dichotomous variable (UST < or > 15 s). The clinical relevance of the research question relates to the point that the more potent the relationship between the three clinical attributes and UST, the more likely the clinician will be able to suggest rational recommendation and/or focused therapy based on specific deficits rather than generic physical therapy referrals to “improve balance.”

## Methods

### Overview

Two prior studies, ongoing research and clinical work from 2016 to the December 2024 served as the sources of data for this observational, cross-sectional study [15, 16]. Institutional Review Board approval was obtained for all three data sources. Age, body mass index (BMI), sex at birth, and number of prescription medications were available for analyses. Additionally, vibratory sensation, reaction accuracy, and lateral plank time, which represented measures of somatosensory precision, central processing speed, and proximal strength, respectively, were also available.

### Participants

Of the 172 participants, 31 had varying degrees of diabetic neuropathy, 94 had varying degrees of cirrhosis without covert hepatic encephalopathy, and 47 were older people without known disorders. Table 1 describes the study participants, who were all functioning independently in the community and did not routinely require assistive devices. People with cirrhosis are particularly relevant for inclusion given their increased rate of falls and the potential for significant consequences as fall-related injury often reduces the likelihood of future transplantation [16]. The participants with cirrhosis were recruited through Hepatology clinic at Michigan Medicine. Inclusion/exclusion criteria included age *≥* 50 and evidence of portal hypertension. Participants were excluded if they were living in a nursing home, experienced binge drinking within the prior 3 months, used a wheelchair, or were hospitalized in the prior 30 days. Of 119 people recruited, 94 had full data sets and were used for this research. The reason for incomplete data were generally lack of time. The 31 participants with diabetic neuropathy were recruited through the Michigan Medicine Electrodiagnostic Laboratory, 26 for research purposes and 5 through clinical participation in a clinical balance evaluation service. Inclusion criteria included age between 50 and 85 years, weight less than 136 kg, known history of diabetes mellitus, and clinical and electrodiagnostic evidence of a distal symmetric polyneuropathy. Potential participants were excluded if there was known central neurologic disease such as Parkinson’s or myelopathy, lower limb joint replacement in the prior year, any lower limb amputation (including digit amputation), or ankle strength less than anti-gravity. Of 56 participants screened, 6 did not pass the physical examination, 8 did not show for appointments or canceled. Of the remaining 42, 26 were people with diabetic neuropathy who were grouped with 5 people with neuropathy in the clinical balance evaluation program. The 16 participants without neuropathy were grouped with 37 evaluated clinically to yield the 53 participants without known disease. All of the participants evaluated clinically produced full sets of data and so there were no dropouts.

**Table 1:** Means and standard deviations for the relevant dependent and independent variables for the whole group and each clinical sub-group

Group	Age %Female	BMI	Number Med	UST^a^ (sec)	VIB^b^ (sec)	RAcc/2^c^ (%)	LPT^d^ (sec)	Comp^e^
All *n* = 172	64.8 ± 9.6 51%	31.2 ± 7.6	8.8 ± 5.8	13.1 ± 10.4	8.2 ± 6.0	32.5 ± 8.6	13.9 ± 11.0	54 ± 17
Cirrhosis *n* = 94	62.5 ± 7.2 46%	31.8 ± 7.9	11.2 ± 6.2	14.1 ± 10.1	9.3 ± 5.2	30.5 ± 8.8	15.5 ± 11.1	55 ± 18
Diabetic Neuropathy *n* = 31	69.3 ± 7.4 44%	32.6 ± 6.3	8.1 ± 2.9	6.2 ± 6.5	3.6 ± 3.9	35.1 ± 7.7	7.8 ± 6.6	46 ± 13
No Disease *n* = 47	66.6 ± 13 67%	29.9 ± 6.5	5.2 ± 4.0	15.5 ± 11.3	9.5 ± 7.2	34.8 ± 7.9	14.4 ± 11.7	59 ± 17

a. UST = Unipedal stance time; b. VIB = Vibration perception; c. RAcc/2 = Reaction Accuracy/2

d. LPT = Lateral plank time; e. Comp = Composite measure

### Dependent variable, unipedal stance time (UST)

Participants initially performed two trials of unipedal stance on the foot of choice, initiating the process with the feet approximately shoulder width apart. Participants were allowed to move their upper limbs as needed but the trial ended if the stance foot shifted or the non-stance foot touched the ground. Then two trials were obtained in the same fashion on the other foot. The mean of the better of the two times, determined with electronic stopwatch, for each foot was used for analyses. Trials were stopped after 30 s of UST.

### Independent variables

#### Age and medication number

Age, body mass index (BMI), sex, and number of prescription medications were used as covariates given their known associations with balance and mobility.

#### Vibratory sensation

Clinical vibratory perception (Vib) of a 128 Hz tuning fork was used as the clinical estimate of foot/ankle proprioceptive precision. A 128 Hz tuning fork was struck and placed on the dorsum of the first digit of the upper limb to allow participant familiarization with perceiving the gradual extinguishing of vibratory sensation and signaling its absence. Then the same procedure was performed placing the fork on the dorsum of each great toe just proximal to the nailbed, and the mean of the two vibration perception times was used for analyses [17]. The resulting value has been shown to detect neuropathy and, more importantly, has demonstrated a strong correlation with a gold standard laboratory measure of ankle inversion/eversion proprioceptive precision [18, 19].

#### Central processing speed

**Fig. 1:**
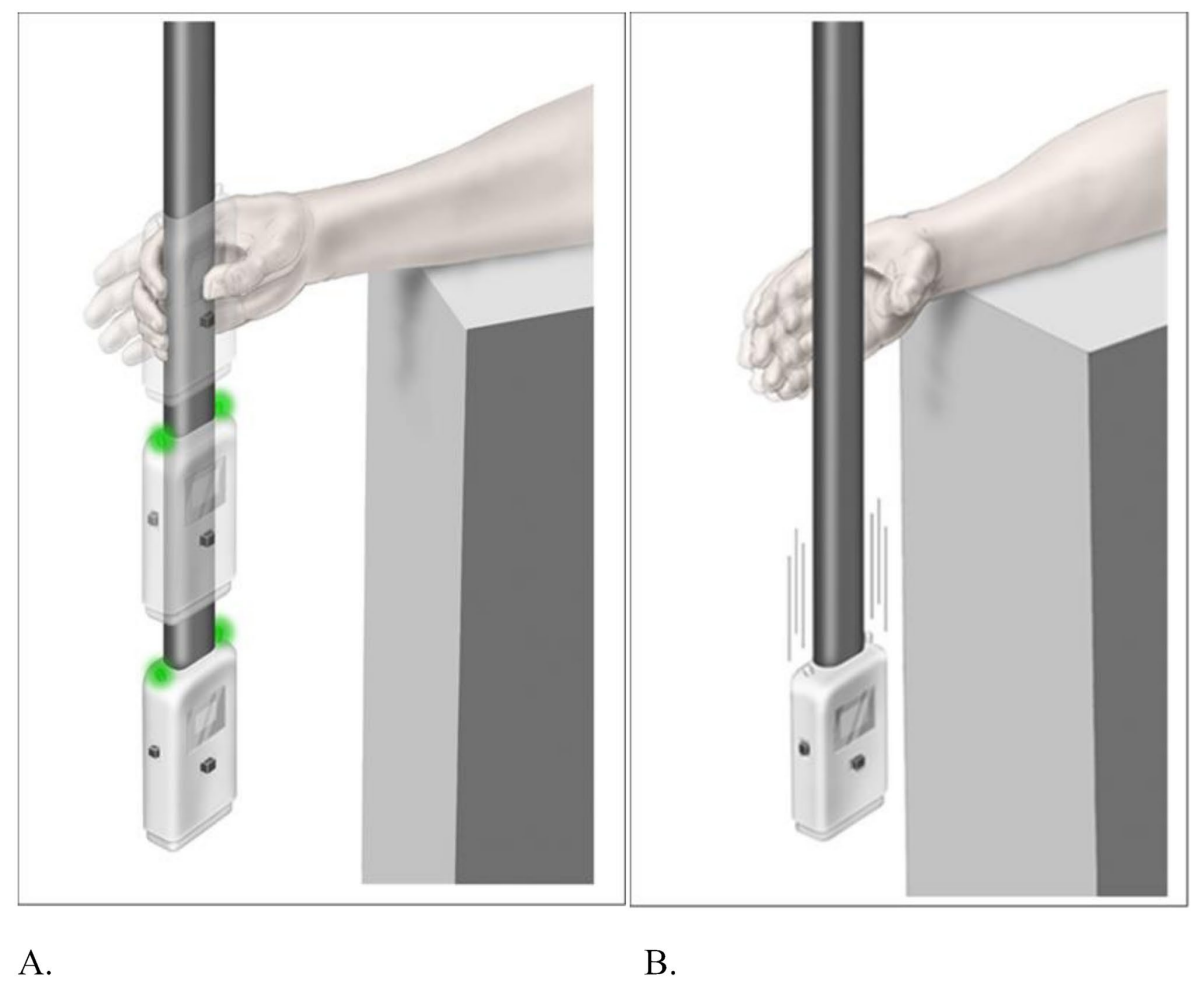
**a.** (left) and **b** (right). Reaction Accuracy (RA) was determined using ReacStick. Initially, simple reaction time (SRT) was evaluated over 10 trials during which the lights atop the housing did not illuminate. The testing mode was then changed, and the lights atop the housing illuminated randomly on 50% of 20 trials. The participant was instructed to catch the device when the lights illuminated (left panel), which occurred at the instant of release, but let the device drop to the floor when the lights did not illuminate (right panel). Instructions clearly emphasized accuracy of response, not speed. The task requires attention, perceptual and motor inhibition, access to working memory (light on = catch; light off = let drop), and excellent processing speed given that execution must occur within 390 milliseconds when released from standard desk height (approximately 31 cm)

ReacStick (See Fig. 1 for full explanation) is a visuo-motor go/no-go measure requiring a response within 390 milliseconds when the device is dropped from desk height. Reaction Accuracy is the percentage of correctly caught/let go responses of 20 trials. ReacStick parameters have shown a potent influence on response to frontal and sagittal perturbations, UST, cautious gait, and grip strength suggesting a strong relationship with functional tasks [15, 20–22]. The latency of response is similar to that required to successfully recover from a perturbation [23].

#### Frontal plane strength

Lateral plank time (LPT) was used as the clinical estimate of hip abductor rate of force generation. The participant laid on their side with hips and knees extended and the downward forearm flat against support surface and the upward arm along the side of the body. The participant was given instructions to elevate the hips to align the trunk and thighs and hold that posture as long as possible. LPT has been shown to be valid and reliable [24, 25]. If too difficult the same maneuver was performed with the knees flexed. The number of seconds elapsed before inability to maintain trunk/thigh alignment was recorded. This value was divided by 2 for knees flexed posture given the greater ease of the task. Rohmert’s law indicates that the residual muscle strength diminishes exponentially in proportion to the time it continuously holds a posture against resistance. A meta-analysis showed trunk endurance time to be a surrogate for trunk power (R^2^ = 0.885) and LPT has shown a significant relationship with laboratory-based frontal plane hip rate of torque generation [19, 26]. Trials were stopped after 30 s with knees extended and after 60 s with knees flexed.

#### Composite variable

To offer clinicians a single number reflecting the three physiologic attributes hypothesized to be essential for UST the following were summed.

- Number of seconds tuning fork was perceived with greater number being associated with more precise foot/ankle proprioceptive precision (range is 0 to 20 s).
- Reaction Accuracy with greater value associated with better short latency processing speed. This value tended to range from 50% (i.e., chance) to 90% (excellent) and was divided in half to render the magnitude of the number (i.e., 25 to 45 for 50% and 90% Accuracy, respectively) in line with the other two variables.
- Number of seconds of Lateral Plank Time with greater number being associated with greater strength (range is 0 to 30).

These variables reflect previously published theory regarding UST and response to pertubation, which is essential to reliable UST [13].

### Statistical analyses

#### UST as a continuous variable

Bivariate Pearson correlation coefficients (r) were generated for age, medication number, BMI, vibratory sense, reaction accuracy, and lateral plank time with UST. Linear regression (SPSS version 26.0) was performed with UST as the dependent variable. Age, BMI, number of prescription medications, and sex were initially put into the model given their known associations with UST. Then the physiological measures were entered into the regression model in the order of vibratory sense, All Accuracy, and LPT. The change in levels of significance and adjusted R^2^ were assessed on the entry of the physiologic variables separately and as a composite variable. Standardized residuals were evaluated for independence and normality. Variance Inflation Factors for each of the three physiologic variables were calculated. A composite variable was then generated for clinical use. This was the sum of vibratory sensation, reaction accuracy (divided by 2 to render it similar in magnitude to the other two variables), and lateral plank time. The composite variable was analyzed in the same manner as described for the three physiologic measures when these were entered separately. The final models only included variables which retained their significance after entry of the three physiologic measures separately or as a composite.

#### UST as dichotomous variable (≥ or < 15 s)

A receiver operator characteristics (ROC) curve was generated using the composite variable and UST dichotomized as greater or less than 15 s.

## Results

### Participant characteristics

Table 1 demonstrates means and standard deviations for the physiologic predictor variables and the outcome variable, UST. Although clinical group differences were not analyzed, as this was not the purpose of the study, inspection of the variables is consistent with what would be expected. For example, the participants with neuropathy had the briefest vibration perception time, the cirrhosis group had the worst reaction accuracy, and the no known disease participants had the highest composite scores.

### Evaluating UST as a continuous measure

#### Bivariate analyses

Pearson correlation coefficients between each of the continuous demographic and independent variables and UST for the entire group were all significant, but not for each of the sub-groups (Table 2). The relationship between the composite variable (vibration time + reaction accuracy/2 + lateral plank time) and UST was strong for the entire group and for each of the three clinical groups evaluated separately.

**Table 2:** Pearson and correlation coefficients between UST and age, BMI, number of medications, and each of the physiologic variables, as well as the composite physiologic variable (vibration time + reaction accuracy/2 + lateral plank time) for each of the clinical groups and for the entire group

	UST			
	All Groups *n* = 172 (r/p)	Neuropathy *n* = 31 (r/p)	Cirrhosis n -= 94 (r/p)	No Disease *n* = 47 (r/p)
Age	− 0.288/<0.001	− 0.435/0.015	− 0.195/0.059	− 0.268/0.068
BMI	0.200/0.013	0.727/0.124	− 0.177/0.056	− 0.475/0.007
Med^a^	− 0.343/<0.001	0.221/0.267	− 0.343/<0.001	0.581/<0.001
VIB^b^	0.432/<0.001	0.520/0.003	0.198/0.055	0.524/<0.001
All Acc^c^	0.427/<0.001	0.471/0.007	0.605/<0.001	0.294/0.045
LPT^d^	0.715/<0.001	0.594/<0.001	0.678/<0.001	0.726/<0.001
Comp^e^	0.807/<0.001	0.775/<0.001	0.775/<0.001	0.834/<0.001

(a) Med = Medication Number; (b) VIB = Vibration perception; (c) All Acc = All Accuracy; (d) LPT = Lateral plank time; (e) Comp = Composite variable

#### Multivariable analyses

**Fig. 2:**
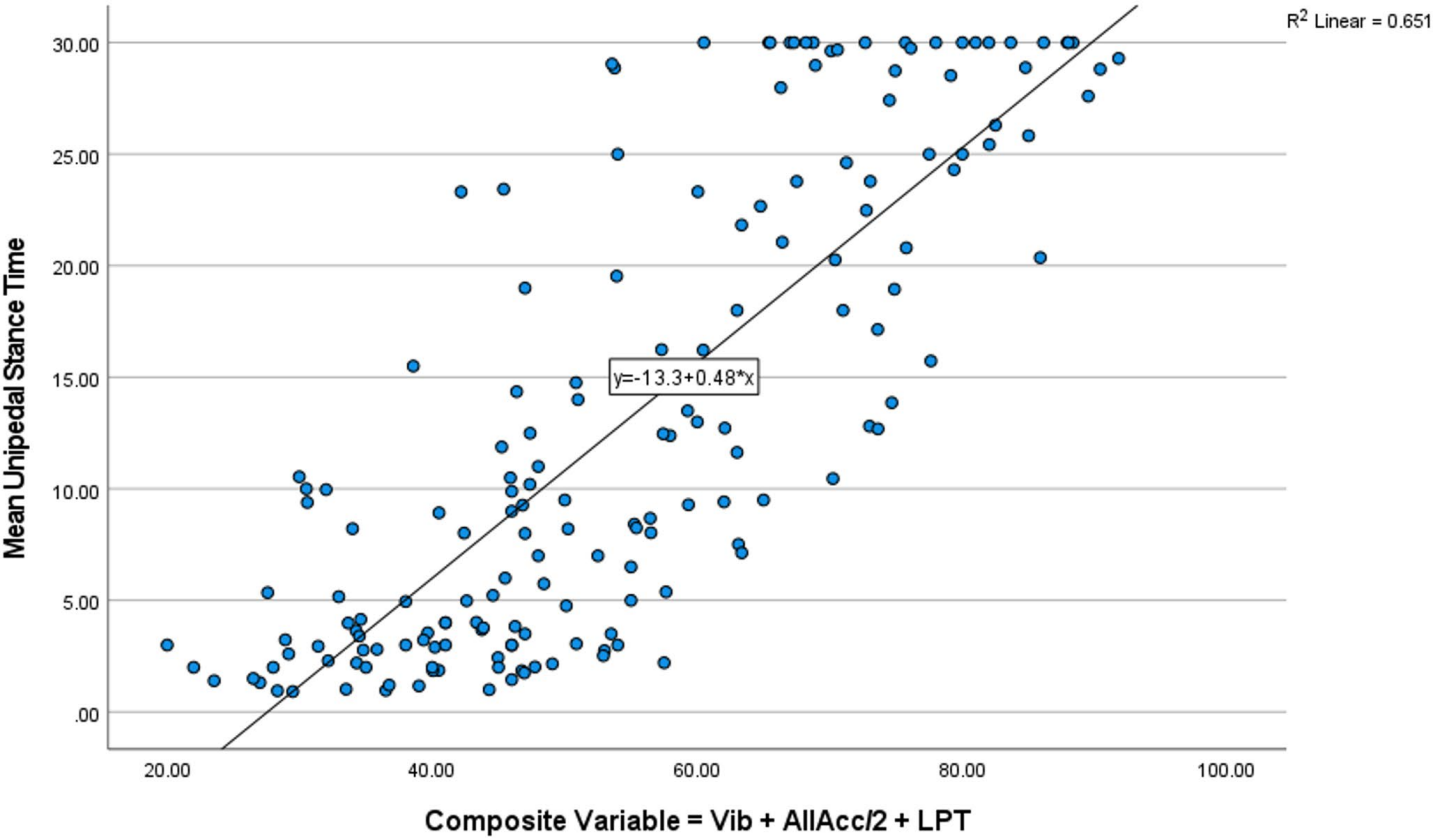
Scatter plot of the composite variable and UST for all groups combined (*n* = 172). Pearson correlation = 0.807/*p* < .001; R^2^ = 0.65

Multivariate analyses demonstrated that the four demographic variables (age, sex, BMI, and number of medications) were all significantly related to UST (*p* values = < 0.001, 0.010, 0.002, and < 0.001, respectively) with the resultant adjusted R^2^ = 0.205. However, when the three physiologic measures were then introduced into the model all of the demographic variables became insignificant (*p* values = 0.346, 0.832, 0.379, and 0.150, respectively) and the adjusted R^2^ = 0.598. The demographic variables were then removed and the three physiologic variables explained about 2/3 of the variability in UST (adjusted R^2^ = 0.659) (Table 3). The same procedure was performed for the composite variable and when it was added the demographic variables again became insignificant (*p* = .526, 0.161, 0.637, and 0.129, respectively) and the adjusted R^2^ = 0.598. The demographic variables were then removed from the model and the resultant adjusted R^2^ = 0.649. (Fig. 2). The composite variable also explained 59, 0.60, and 0.68 of UST for each sub-group (neuropathy, cirrhosis, and no disease, respectively). Of note, the variance inflation factors for Vib, AllAcc, and LPT were 1.036, 1.093, and 1.056, respectively indicating that adjustments to the final regression models were not necessary.

**Table 3:** Results of linear regression with UST as the dependent variable and vibratory sensation, reaction accuracy, and lateral plank time entered as independent variables. The composite variable (vibratory sensation + reaction accuracy/2 + lateral plank time) also significantly predicted UST with minimal change in the adjusted R^2^

Variable	95% CI^g^						
	Beta^e^	SE^f^	LL^h^	UL^i^	β^j^	ƿ	Adjusted R^2^
Vib^a^	0.53	0.079	0.38	0.69	0.31	< 0.001	0.182
All Acc^b^	0.35	0.057	0.23	0.46	0.28	< 0.001	0.349
LPT^c^	0.55	0.044	0.47	0.64	0.58	< 0.001	0.659
Composite^d^	0.48	0.027	0.43	0.54	0.81	< 0.001	0.649

a. Vib = Vibration perception; b. All Acc = All Accuracy; c. LPT = Lateal plank time; d. = Composite variable; e. Beta = Unstandardized beta values; f. SE = Standard error; g. CI = Confidence interval; h. LL = Lower limit; i. UL = Upper limit; j. β = Standardized beta weights provided give an indication of the relative importance of physiologic measures entered into the model explaining variance in UST times

### Evaluating UST as a dichotomous measure

The composite variable ROC curve with UST < 15 s considered the diagnostic category of interest resulted in area under the curve (AUC) of 0.932 (95% CI = 0.88 to 0.97) suggesting excellent discrimination. A composite variable cut-off of 53 yielded a sensitivity of 0.93 and a specificity of 0.77 for identifying participants with UST < 15 s. The same AUC for each of the sub-groups was 0.972 (95% CI = 0.914, 1.00), 0.891 (95% CI = 0.826, 0.957), and 0.958 (95% CI = 0.902, 1.00), for the people with diabetic neuropathy, cirrhosis, and no known disease. Optimal cut-points were 59, 53, and 54, respectively. Additionally, the AUC’s for people younger than 65 and 65 or older were 0.890 (95% CI = 0.82, 0.959) and 0.952 (95% CI = 0.904, 0.999), respectively with optimal cut-points of 53 and 54. These sub-group analyses suggest that the composite variable demonstrated similar discriminatory capacity across clinical groups.

## Discussion

Our analyses suggest that peripheral afferent function (vibration sense), central processing speed (reaction accuracy), and frontal plane strength (lateral plank time), predict about two thirds of the variability in UST. (Table 2). This has clinical relevance given recent findings that UST < 15 and < 10 s predicts repetitive falls and mortality, respectively, 5 to 10 years later. It is well documented that UST declines with age and age-adjusted norms for UST [27] are available. Importantly, when the three physiologic variables were accounted for age, BMI, sex, and number of medications had no influence on UST. This suggests that these variables are not inherently a detrimental to balance but serve as markers for less precise distal somatosensory function, delayed processing speed, and/or diminished force generation in key muscles which are the true reasons for the age-related decline in UST, and apparent adverse effects of BMI and number of medications. Although the composite variable did not explain quite as much of the variance in UST as did the three variables considered separately, the difference was 1% (Adj R^2^ = 0.649 for composite variable vs 0.659 for three individual variables) which is negligible. Additionally, the composite offers an intuitive advantage for clinicians who are aware of poor vs. robust measures: For Vib, 0 s is poor while 15 s at the toe is highly sensitive; poor All Accuracy not better than chance is 50% while 90% is excellent; and LPT of 0 s is poor while 30 s is strong. Using these variables in their unchanged form allows the clinician to develop a perspective on patient strengths and weakness which leads to rational therapy in real time as the evaluation proceeds. Given these, the composite variable has potential for clinical utility when patients with poor balance are identified.

Why were these clinical attributes successful in predicting UST? Given that sub-clinical physiological declines in sensation, strength, reaction time, and processing speed occur during middle age [28], a key point in the work presented here is that the clinical estimates of the relevant physiologic attributes were considered in a semi-quantitative manner which served to stratify even high performers. Note that in Fig. 2 few achieved a composite score > 85 (e.g., Vib 15 s, AllAcc 90%, and LPT 25 s). This contrasts with some examination methods which consider, for example, vibratory sense to be present or absent, or insufficiently differentiate between people at higher ends of strength or cognitive function. And yet these typically unaccounted for decrements in key physiologic attributes exist in the middle years. For example, isometric and dynamic knee extensor muscle strength in men aged 50–65 years is 20–35% less than that of men aged 20 to 35 years [29], and muscle strength in middle aged women (aged 42–61 years) is reduced by 5–23% as compared to young women [30]. Women in their 40’s and 50’s demonstrate decrements in somatosensory function, in addition to poorer strength, as compared to younger women and these decrements are linked to reduced UST [31]. Finally, it is well known that declining cognition, particularly executive function, adversely affects balance and increases fall risk. Importantly, there is evidence that executive function is inferior in people in their late 50’s compared with those younger than 35 [32]. Further, it appears that these declines are greater in the middle years in people with metabolic syndrome [33] and less marked in those who are physically active [34]. The challenge is to identify cognitive changes in the middle years, when there is a departure from optimal cognition, rather than waiting to initiate treatment after falls are taking place.

The link between the clinical estimates used and their respective physiologic attributes deserves comment. Vibratory sensation is known to stimulate mechanoreceptors (Pacinian and Meissner corpuscles) which then transmit through the posterior column-medial lemniscal pathway. These receptors tend to be located in hairless skin and so likely contribute, along with specialized receptors in muscles and joints, to proprioceptive precision through the same central pathway [35]. Therefore, vibratory sensation appears to measure one aspect of proprioception and uses the same central pathway, and these features allow vibration to be a reasonable clinical estimate of proprioceptive precision. In prior work, Reaction Accuracy which is a go/no-task within 390 milliseconds was found to relate to grip strength whereas a computerized go/no-go task which allowed 2000 milliseconds to respond did not [22]. Given that grip strength is known to relate to central white matter integrity, it is likely that Reaction Accuracy is a clinical surrogate for central white matter integrity, which strongly influences gait and postural stability [36]. Lastly, LPT exploits Rohmert’s law which states that residual muscle strength diminishes exponentially in proportion to the time it continuously holds a posture against resistance. Accordingly, LPT is a reasonable clinical surrogate for trunk power (R^2^ = 0.885) and LPT has shown a significant relationship with laboratory-based frontal plane hip rate of torque generation [19, 26]. In summary, each of the clinical measues has a clear relationship with its associated physiologic capacity critical to UST.

The ability to determine why, specifically, a patient in their middle years cannot perform UST for > 15 s, a cut-off which predicts increased risk for repetitive falls 10 to 12 years later [3], offers the possibility of a novel approach to falls prevention through early intervention. This stands in contrast to the 2022 World Guidelines for Falls Prevention and Management which emphasize employing fall-reduction strategies in those already demonstrating gait and balance problems [37]. Similarly, a recent systematic review of 198 recommendations across 15 selected guidelines recommended using risk stratification based on “fall history, fear of falling, and gait and balance difficulties, and restricting gait and balance testing to only those who screen positive on those questions” [38]. While focusing on those at high risk of further falls has undoubted benefits, we suggest a complementary strategy which would initiate targeted fall prevention strategies for people who cannot perform 15 s UST in their middle years, in other words before falls occur. The findings presented here can serve to guide potential interventions.

There are several possible strategies for addressing the specific physiologic impairment(s) responsible for diminished UST. Interventions designed to improve trunk extensor strength in older people have a laudatory effect on UST control and duration [39]. Hip abduction strength is also critically important for reliable UST, as noted in biomechanical and clinical studies [7, 40]. In support, post-menopausal women (mean age 58 years) improved UST following a high intensity water exercise regimen [41]. If strength cannot increase, weight loss can instead serve to increase functional strength which may translate to improved UST, consistent with prior work correlating increased body mass index with diminished UST [42]. Plantar sensation influences UST and mechanical noise to the plantar aspect of the feet improves bipedal balance and gait in some populations, offering this intervention as a possibility [43]. Vision appears important for UST given data showing that sighted people have better UST than blind people, despite the latter demonstrating more precise ankle proprioceptive precision [44]. Additionally, optimizing vision appears necessary given that visual acuity and depth perception relate to performance in challenging standing balance tasks [45]. Lastly, hip strength appears to compensate for diminished peripheral sensation allowing greater UST [6].

One of the most clinically feasible interventions to improve processing speed is the removal of unnecessary medications, particularly those with anti-cholinergic potential which are known to influence executive functions and fall risk [46, 47]. Additionally, insufficient sleep and sleep quality degrade executive function and motor coordination and should be evaluated in people with poor balance [48]. Anxiety and depression are common, treatable clinical conditions also associated with reduced processing speed and mobility function [49]. Chronic pain adversely affects UST, and this appears to be mediated by cognitive processing speed [50]. In addition to addressing these conditions which hinder optimal short latency cognitive function, a review suggests that physical activity has a significant impact on executive functions and, possibly, processing speed in middle-aged adults [34] and is associated with reduced degrees of cerebral white matter degradation in older adults [51]. 1Another review indicates that a holistic approach to improving executive function is indicated given that stress, sleep disorders, social isolation, and insufficient exercise each lead to executive impairments [52]. Prospective studies are needed to determine whether these interventions can improve UST.

Among this paper’s strengths are the robust relationships between the three variables and UST, and the highly similar relationships seen across three heterogeneous clinical populations. However, limitations must also be recognized. Although vibratory sense and reaction accuracy are known to have reasonable inter-rater and re-test reliabilities [18, 53], and the reliability of LPT has been evaluated in younger adult athletes and younger adults with multiple sclerosis [24, 25], it has not been evaluated in older adults. Further, the technique of evaluating LPT with knees flexed, when performing the task with knees extended, has also not been proven reliable in this population, although the validity of the method has support [19]. LPT likely relies on truncal strength as well as hip abduction/adduction strength but does not account for frontal plane ankle strength, which represents another limitation to our methods. In addition, there was no formal sample size determination and the number of people studied was moderate, and the majority had a disorder expected to impact the physiologic variables analyzed. This may have increased the range of data allowing stronger correlations than might result if evaluating a generally healthy people in their middle years. However, the people tested may have not been excessively dissimilar from clinic populations commonly seen given that the prevalence of metabolic syndrome, which is associated with neuropathy, diminished strength and executive functions, is approximately 50% in people over 60 in the U.S. and estimated to be about 25% worldwide regardless of age [54, 55].

## Conclusion

In conclusion, this paper offers evidence that three clinically feasible attributes which estimate critical physiologic capacities predict two-thirds of UST variation, and that when these attributes are accounted for age itself does not influence UST. The findings suggest that age in isolation is not a risk factor for diminished UST, but is a marker for subtle, sub-clinical decrements in key physiologic capacities which are the true source of age-related postural instability. The findings are of clinical relevance given recent evidence that diminished UST in the middle years predicts repetitive falls and mortality 5 to 10 years later. Clinical evaluations of these specific capacities allows the development of patient specific therapeutic interventions rather than generic physical therapy referrals to “improve balance.” This strategy may offer a complementary approach to fall prevention which currently primarily focuses on falls after gait changes are manifest and falls are occurring. Further research is needed to validate these findings in larger and more diverse populations and, more importantly, to explore the effectiveness of targeted interventions based on these measures.

## Data Availability

No datasets were generated or analysed during the current study.
